# A randomized double-blind study of testosterone replacement therapy or placebo in testicular cancer survivors with mild Leydig cell insufficiency (Einstein-intervention)

**DOI:** 10.1186/s12885-017-3456-5

**Published:** 2017-07-03

**Authors:** Mikkel Bandak, Niels Jørgensen, Anders Juul, Jakob Lauritsen, Michael Kreiberg, Peter Sandor Oturai, Jørn Wulff Helge, Gedske Daugaard

**Affiliations:** 1 Department of Oncology, Copenhagen University Hospital, Rigshospitalet, 2100 Copenhagen, Denmark; 2 Department of Growth and Reproduction, Copenhagen University Hospital, Rigshospitalet, Copenhagen, Denmark; 3 International Center for Research and Research Training in Endocrine Disruption of Male Reproduction and Child Health (EDMaRC), University of Copenhagen, Copenhagen, Denmark; 4 Department of Clinical Physiology, Nuclear Medicine and PET, Copenhagen University Hospital, Rigshospitalet, Copenhagen, Denmark; 5 Department of Biomedical Sciences, University of Copenhagen, Copenhagen, Denmark

**Keywords:** Testicular cancer, Mild Leydig cell insufficiency, Testosterone substitution

## Abstract

**Background:**

Elevated serum levels of luteinizing hormone and slightly decreased serum levels of testosterone (mild Leydig cell insufficiency) is a common hormonal disturbance in testicular cancer (TC) survivors. A number of studies have shown that low serum levels of testosterone is associated with low grade inflammation and increased risk of metabolic syndrome. However, so far, no studies have evaluated whether testosterone substitution improves metabolic dysfunction in TC survivors with mild Leydig cell insufficiency.

**Methods/design:**

This is a single-center, randomized, double-blind, placebo-controlled study, designed to evaluate the effect of testosterone replacement therapy in TC survivors with mild Leydig cell insufficiency. Seventy subjects will be randomized to receive either testosterone replacement therapy or placebo. The subjects will be invited for an information meeting where informed consent will be obtained. Afterwards, a 52-weeks treatment period begins in which study participants will receive a daily dose of transdermal testosterone or placebo. Dose adjustment will be made three times during the initial 8 weeks of the study to a maximal daily dose of 40 mg of testosterone in the intervention arm. Evaluation of primary and secondary endpoints will be performed at baseline, 26 weeks post-randomization, at the end of treatment (52 weeks) and 3 months after completion of treatment (week 64).

**Discussion:**

This study is the first to investigate the effect of testosterone substitution in testicular cancer survivors with mild Leydig cell insufficiency. If positive, it may change the clinical handling of testicular cancer survivors with borderline low levels of testosterone.

**Trial registration:**

ClinicalTrials.gov: NCT02991209 (November 25, 2016).

## Background

Testicular cancer (TC) is the most common solid tumour in men between 18 and 35 years of age in developed countries [1], and more than 95% of patients become long-term survivors [2]. In consequence, a wide range of studies have investigated the prevalence of late effects to TC treatment such as hormonal disturbances [3–5], cardiovascular disease [6–8] and metabolic syndrome [9–12]. The risk of cardiovascular disease is increased in TC patients treated with cisplatin-based chemotherapy when compared to the background population, and it has uniformly been reported that low serum levels of testosterone are associated with increased risk of metabolic syndrome. This has led to the hypothesis that decreased serum levels of testosterone in cancer survivors increase the risk of insulin resistance, metabolic syndrome and long-term risk of cardiovascular disease [13]. Subjective symptoms of testosterone deficiency are decreased libido, decreased level of energy, depressive symptoms, impaired cognitive function and decreased muscle strength while objective signs are decreased bone density, decreased lean body mass, abdominal obesity and anemia [14, 15]. It is well documented that testosterone replacement therapy improves these conditions in patients with *manifest testosterone deficiency* [16, 17] and numerous studies have demonstrated increased insulin sensitivity, decreased systemic inflammation and recovery of components of the metabolic syndrome [18–20]. However, a substantial proportion of TC survivors are in a compensated state with elevated luteinizing hormone (LH)-levels in combination with borderline low levels of testosterone (*mild Leydig cell insufficiency*) [21]. To our knowledge, only one study has examined the effects of testosterone replacement therapy in mild Leydig cell insufficiency [22]. This randomized, placebo-controlled study investigated the effect of 12 months of testosterone replacement therapy in 30 long-term survivors of haematological cancer and found a reduction of fatigue and a slight decrease in low density lipoprotein (LDL) cholesterol, and concluded that testosterone substitution should not be recommended in this condition. However, the limit for testosterone replacement therapy was total testosterone <20 mmol/L and LH > 8 IU/L and the effect on fasting blood glucose, insulin sensitivity and metabolic syndrome was not evaluated.

Thus, the purpose of the present study is to determine whether testosterone replacement therapy improves insulin sensitivity and thus potentially reduces the long-term risk of cardiovascular disease in TC survivors with mild Leydig cell insufficiency after TC treatment.

## Methods/design

### Study subjects

Seventy TC survivors with no relapse >1 year since treatment will be included in the study. The main inclusion criteria are serum levels of LH > 2 standard deviations (SD) above the age-adjusted mean value and serum levels of free testosterone between 2 SD below the age-adjusted mean value and the age-adjusted mean value. Further inclusion and exclusion criteria are presented in Table 1.

**Table 1:** Eligibility Criteria

*Inclusion criteria* - Age > 18 years and <65 years - Previous treatment for testicular cancer - No signs of relapse 1 year after the last treatment (orchiectomy, radiotherapy, chemotherapy) - Serum free testosterone < the age-adjusted mean value and >2 standard deviations (SD) below the age-adjusted mean value - Serum luteinizing hormone (LH) > 2 SD above the age-adjusted mean value
*Exclusion criteria* - Testosterone treatment within the last 6 months - Contraindications to testosterone treatment: (prostate cancer, prostate specific antigen (PSA) > 4 ng/mL), malignancy suspect prostate by digital rectal examination (ALT) > 1.5 upper limit of normal, erythrocyte volume fraction (EVF) > 50%, breast cancer - Symptomatic obstructive sleep apnoea syndrome - Heart failure > New York Heart Association class II - Uncontrolled hypertension: (Systolic blood pressure > 160 mmHg despite antihypertensive treatment, measured at two separate occasions) - Inability to understand information about the trial - Participation in any other clinical trial - Allergy for the active substance or additives in Tostran or placebo. - Known diabetes mellitus, or diabetes mellitus detected at screening or at baseline - Paternity wish at the time of inclusion^a^

^a^If there is any doubt about paternity wish at the time of inclusion, the study subject should give a semen sample for analysis. If the semen sample shows any viable sperm cells, the study subject will be excluded

### Recruitment and informed consent

**Fig. 1:**
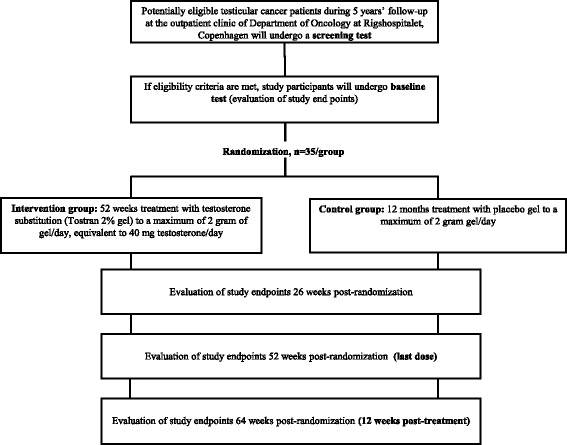
Study flow diagram

The study flow is presented in Fig. 1. Potential study subjects will be recruited by clinicians during 5-years follow-up after treatment for TC at the outpatient clinic of Department of Clinical Oncology at Copenhagen University Hospital, Rigshospitalet. Interested patients will be contacted by the study coordinator, who will provide oral and written information about the study. An informed consent form will be signed, when there is a mutual understanding that the subject has understood the information given and still wants to participate. Afterwards, an appointment for screening will be made.

### Randomization and procedures for breakage of randomization code

The 70 study participants will be randomized 1:1 to active treatment or placebo. Randomization will be done by a web-based randomization tool (http://www.randomization.com). Each subject will be assigned a specific and unique randomization number. The randomization code will be broken for a subject if a suspected adverse event is judged by the investigators, as serious as it will influence future treatment. The randomization code can be broken at any time during the study period.

### Blinding

Study participants and primary investigators are blinded. An unblinded investigator will evaluate safety issues.

### Intervention

If a subject meets the inclusion criteria, reviewed at the screening and after the baseline investigations, randomization will take place and the study participant will be allocated to treatment with testosterone replacement therapy or placebo. He will initiate treatment the following day in the morning.

*Study arm 1: Testosterone (Tostran 2% gel).*

Study participants allocated to active treatment will receive 12 months daily treatment with Tostran 2% applied transdermally. Dose adjustment will take place three times during the initial 8 weeks to a maximal daily dose of 40 mg (four depressions of the piston) (Table 2).

**Table 2:** Dose adjustments and dose modifications due to safety issues

Dose adjustment	Dose modifications	Follow-up
Starting dose: 10 mg daily (one depression of the piston)		
Week 2: 20 mg daily (two depressions of the piston)		
Week 4: 30 mg daily (three depressions of the piston)		
Week 8: 40 mg daily (four depressions of the piston)		
52 weeks: End of treatment		
Safety issues		
Increase in free testosterone to >3 SD above the age-adjusted mean	Dose adjustment to previous treatment step	Measurement of free testosterone and LH after 14 days and dose adjustment accordingly
Decrease in LH to < −2 SD below the age-adjusted mean	Dose adjustment to previous treatment step	Measurement of free testosterone and LH after 14 days and dose adjustment accordingly
Increase in EVF to >52%	Dose adjustment to previous treatment step	Measurement of EVF after 14 days and dose adjustment accordingly
Plasma PSA > 4 ng/mL at any visit. A PSA increase of >1 ng/mL at visit 7 or visit 8, using plasma PSA at visit 6 as baseline	Referral for urological consultation	According to urological consultation: Stop treatment if increased risk of prostate cancer. Continue treatment at the same treatment step if there is no increased risk of prostate cancer
An increase in systolic blood pressure > 20 mmHg confirmed at two separate measurements despite antihypertensive therapy, if not due to free testosterone being >3 SD from the age-adjusted mean	Stop treatment	Measurement of blood pressure after 14 days. If still elevated referral to general practitioner
ALT increase >1.5 upper reference level	Stop treatment	Measurement of ALT after 14 days. If still elevated referral for hepatological consultation
Any other exclusion criteria becoming apparent during treatment	Stop treatment	

*Study arm 2: Placebo.*

Study participants allocated to placebo will receive 12 months daily treatment with placebo. Identical dose adjustment will take place during the initial 8 weeks to four depressions of the piston (Table 2).

The summary of product characteristics of Tostran 2% can be seen at (http://www.medicines.org.uk/emc/medicine/19702). Placebo contains the same substances as Tostran 2% except for testosterone.

Tostran 2% and placebo will be supplied in an identical container labelled with a unique number. Each container has a piston and a depression of the piston delivers 0.5 g of gel which is equivalent to 10 mg of testosterone in the intervention group.

### Dose modifications

Dose adjustment during the initial 8 weeks and criteria for dose modifications due to safety issues are shown in Table 2. If dose modification due to safety issues is needed, a study participant treated with placebo of the same age or age closest to the study participant who should be dose adjusted will be chosen for identical dose modification to keep the double-blinded study design. If more than one placebo-treated patient is at the same age, the subject with a BMI closest to the study participant who should be dose-adjusted will be chosen for identical dose modification.

### Planned inclusion

It is estimated that 70 study participants will be included by the end of 2018.

## Measurements

The schedule for data assessment is presented in Table 3 and the study endpoints are presented in Table 4.

**Table 3:** Data Assessment Schedule

Data assessment	Screening	Baseline Week 0	Dose adjustment 1 Week 2	Dose adjustment 2 Week 4	Dose adjustment 3 Week 8	On treatment Week 26	Last dose Week 52	Follow-up Week 64
hCG-stimulation test	x							
Blood pressure	x	x	x	x	x	x	x	x
Blood samples	x	x	x	x	x	x	x	x
Waist circumference		x				x	x	x
DXA scan		x				x	x	x
Oral glucose tolerance test		x				x	x	x
Questionnaires		x				x	x	x
Anogenital distance		x					x	
Randomization		x						
On treatment		x	x	x	x	x	x	
Dose adjustment			x	x	x			
Evaluation of side effects		x	x	x	x	x	x	x

**Table 4:** Study Endpoints

*Systemic effects* - Glucose metabolism (fasting glucose, 2 h glucose, insulin, hemoglobin a1c, HOMA-index) - Cholesterol (high density lipoprotein-cholesterol, low density lipoprotein-cholesterol, total cholesterol) - Inflammatory markers (interleukin 1-β, interleukin-6, interleukin-8, tumour necrotic factor-alpha) - Adipocytokines (adiponectin, leptin) - Reproductive hormones (total testosterone, free testosterone, luteinizing hormone)
*Body composition and bone mineral density* - Whole body bone mineral density, whole body t-score, t-score of the lumbar spine and proximal femoral bone - Lean body mass - Whole body fat percent (Z-score) - Visceral adiposity, android fat distribution, gynoid fat distribution
*Anthropomorphic measures* - Waist circumference - Hip circumference - Body mass index - Anogenital distance
*Patient reported outcomes* - Health related quality of Life (EORTC QLQ-C30) - Anxiety and Depression: (Hospital Depression and Anxiety Scale) - Fatigue: (Multiple Fatigue Inventory-20) - Symptoms of testosterone deficiency and erectile dysfunction: (International Index of Erectile Function-15)

*Abbreviations*: *HOMA-index* Homeostasis model assessment index, *EORTC QLQ-C30* European Organization for Research and Treatment of Cancer Quality of Life Questionnaire

A human chorionic gonadotropin (hCG) stimulation test will be done at the screening visit in order to evaluate the residual capacity of the remaining testicle in the included study participants: After blood sampling, where serum testosterone is analysed, 5000 IU of hCG is injected intramuscularly. Blood samples are drawn 72 h after the administration of hCG for evaluation of serum testosterone.

Study endpoints will be evaluated at baseline (before randomization), 26 weeks post-randomization, 52 weeks post-randomization (last dose) and 64 weeks post-randomization (12 weeks after last dose). Participants will arrive at the hospital after an overnight fast (minimum 8 h) at visits where study endpoints will be evaluated.

### Primary outcome

The primary endpoint is insulin sensitivity expressed as the 2 h change in blood glucose (∆2 h glucose) evaluated by oral glucose tolerance test: Fasting blood samples will be drawn where plasma glucose and insulin are analysed. Afterwards, at time zero, 75 g glucose will be administered orally after being dissolved in 250–300 ml water. Plasma glucose and insulin will be analysed again after 2 h.

### Secondary outcomes

Evaluation of each component of *metabolic syndrome* (waist circumference, blood pressure, fasting plasma glucose, plasma HDL-cholesterol and plasma triglycerides) as well as presence of metabolic syndrome (≥3 components) will be assessed according to the International Diabetes Federation criteria [23]. Waist circumference and hip circumference will be measured according to guidelines from World Health Organization [24] and blood pressure will be determined twice with an automatic device after 5 min rest.

Plasma samples for analysis of *inflammatory markers:* tumor necrotic factor-alpha (TNF-alpha), interleukine (IL)-1β, IL-6 and IL-10 as well as *adipocytokines* (adiponectin and leptin) will be processed and stored at −80 C° for batch analysis after completion of the study. Inflammatory markers will be analysed by MesoScaleDiscovery multiplex (MesoScaleDiscovery, USA) while leptin will be analysed by immunoassay and adiponectin by radioimmunoassay.

Total plasma cholesterol, HDL-cholesterol, LDL-cholesterol, plasma glucose, haemoglobin A1c and plasma insulin will be measured using standard laboratory procedures. Homeostasis Model Assessment index will be calculated as described in [25].

Serum total testosterone and free testosterone will be analysed by liquid chromatography–mass spectrometry and LH will be analysed by time-resolved immunofluorometric assay (Delfia; Perkin Elmer, Turku, Finland),

*Body composition and bone mineral density* will be assessed by whole body Dual-energy X-ray absorptiometry (DXA-scan) performed on a Lunar Prodigy Advance Scanner (GE Healthcare, Madison, WI; software package Encore v. 16). Whole body bone mineral density (BMD) will be determined, as well as BMD of the lumbar spine and proximal femoral bone. Lean body mass, whole body fat percent, and visceral fat mass will be determined.

Anogenital distance will be measured in supine position with the legs abducted allowing the soles of the feet to meet as described in [26]. The anogenital distance will be measured at baseline and 52 months post-randomization using a digital caliper.

*Patient reported outcomes* will be assessed by standardized questionnaires: Quality of life (EORTC QLQ-30) [27], fatigue (Multiple Fatigue Inventory) [28], symptoms of testosterone deficiency and erectile dysfunction (International Index of Erectile Dysfunction) (IIEF-15) [29] and depression and anxiety (Hospital Anxiety and Depression Scale) (HADS) [30].

EORTC QLQ-30 consists of nine symptom scales, five function scales, and a global health/quality of life scale. A high score on the symptom scales indicates a high symptom burden while a high score on the function scales and global function scale indicates lower symptom burden. MFI-20 is covering five domains of fatigue: general fatigue, physical fatigue, reduced activity, reduced motivation and mental fatigue. A higher score on each of the five scales indicates more fatigue. HADS consists of two subscales covering anxiety and depression. A higher score on each scale indicates higher symptom burden. IIEF-15 examines the five main domains of male sexual function: Erectile function, orgasmic function, sexual desire, overall satisfaction and intercourse satisfaction. A lower score indicates higher symptom burden.

Additionally, study participants will be asked about medication, tobacco consumption, alcohol consumption and level of physical activity.

### Adverse events

Presence of side effects according to common toxicity criteria version 4.0 (https://evs.nci.nih.gov/ftp1/CTCAE/CTCAE_4.03_2010-06-14_QuickReference_5x7.pdf) will be evaluated at each visit and documented in case report forms.

### Sample size

It is expected that 30 study participants in each arm will complete the study to week 52. A power calculation has been conducted and with 30 study participants in each arm, there is 80% power to show a 0.5 mmol difference in ∆2 h glucose between baseline and 52 weeks between the two arms. The significance level is set at 5%.

### Analytic plan

Changes in continuous outcomes between baseline and 52 weeks will be compared between the investigational group and placebo group with independent samples *t*-test. Changes in prevalence of metabolic syndrome components and metabolic syndrome between baseline and 52 weeks will be compared with chi-square test. Changes in patient reported outcomes and adverse events between baseline and 52 weeks will be compared with Mann–Whitney U-test. As an additional analysis outcomes in the intervention arm will be compared with independent samples t-test and chi-square test between study participants who had an adequate response to the hCG stimulation test performed at the screening visit and those who had and inadequate response.

## Discussion

Testosterone substitution is currently advocated in men with evident biochemical testosterone deficiency measured on >1 occasion in concordance with clinical signs and symptoms of testosterone deficiency in the general population [31]. Mild Leydig cell insufficiency is a common condition in testicular cancer survivors, and it has not been clarified whether testosterone substitution is of potential benefit in this population. The results of the present study might have the potential to change the clinical handling of testicular cancer survivors with mild Leydig cell insufficiency in order to decrease the long-term risk of cardiovascular disease.

Recruitment of study participants began in November 2016. By the end of January 2017, approximately 60 potential study participants have received information about the study of which 22 have signed informed consent and been through screening examinations. The most common reason for not wanting to participate in the study after having received information has been that potential study participants found the study too time consuming. Fear of adverse effects to testosterone treatment has been the second most common reason for non-paticipation.

Ten of the 22 screened study participants have so far been randomized, four are in line for randomization while there have been eight screening failures.

As testosterone and LH show considerable day –to-day variation [32] screening failures would be expected. It should, however, be emphasized that all study participants who are undergoing screening examinations have experienced elevated serum LH levels in combination with borderline low serum testosterone at a previous point in time. Accordingly, inclusion in this study is based on at least two measurements of elevated LH and borderline low testosterone levels.

In conclusion, the results of this study will provide evidence for the clinical handling of TC survivors with mild Leydig cell insufficiency and potentially improve morbidity and quality of life in this subgroup of cancer survivors.

## Abbreviations

Einstein: Early INtervention with teSTostErone IN in testicular cancer survivors
HDL-cholesterol: High density lipoprotein cholesterol
LDL-cholesterol: Low density lipoprotein cholesterol
LH: Luteinizing Hormone
SD: Standard deviation
TC: Testicular cancer
